# Grapefruit-Derived Vesicles Loaded with Recombinant HSP70 Activate Antitumor Immunity in Colon Cancer In Vitro and In Vivo

**DOI:** 10.3390/biomedicines12122759

**Published:** 2024-12-03

**Authors:** Luiza Garaeva, Elena Komarova, Svetlana Emelianova, Elena Putevich, Andrey L. Konevega, Boris Margulis, Irina Guzhova, Tatiana Shtam

**Affiliations:** 1 St. Petersburg Nuclear Physics Institute Named by B.P. Konstantinov of National Research Centre «Kurchatov Institute», Orlova roshcha 1, Gatchina 188300, Russia; garaeva_laa@pnpi.nrcki.ru (L.G.); emelyanova_ss@pnpi.nrcki.ru (S.E.); putevich_ed@pnpi.nrcki.ru (E.P.); konevega_al@pnpi.nrcki.ru (A.L.K.); 2 Institute of Cytology of Russian Academy of Sciences, Tikhoretsky Ave. 4, St. Petersburg 194064, Russia; elpouta@yahoo.com (E.K.); margulis@incras.ru (B.M.)

**Keywords:** HSP70, extracellular vesicles, plant-derived extracellular vesicles, drug delivery systems, antitumor immunity, colon cancer

## Abstract

**Background/Objectives:** Stress protein HSP70 administered exogenously has demonstrated high potential as an efficient adjuvant in antitumor immune response. To enhance the antigen-presenting activity, bioavailability, and stability of exogenous recombinant human HSP70, we propose incorporating it into plant extracellular vesicles. Earlier, we found that grapefruit-derived extracellular vesicles (GEV) were able to store the protein with no loss of its major function, chaperone activity. **Methods**: In this study, we tested whether HSP70 loaded into GEV (GEV-HSP70) could elicit an antitumor immune response in cellular and animal models of colorectal cancer. **Results:** To test the hypothesis in vitro, human and mouse colorectal cancer cell lines were used. We have shown that the addition of HSP70, either in free form or as part of GEVs, increases the sensitivity of human (HCT-116, DLD1) or mouse (CT-26) colon cancer cells to mouse cytotoxic lymphocytes and human NK-92 cells. Moreover, the amount of protein in the form of GEV-HSP70 required to cause the same activation of antitumor immunity was 20 times less than when HSP70 was added in free form. In a colon carcinoma model in vivo, GEV-HSP70 were inoculated subcutaneously into BALB/c mice together with CT-26 cells to form a tumor node. As compared with the control groups, we observed an increase in the lifespan of animals and a decrease in the tumor size, as well as a decrease in the level of TGFB1 IL-10 factors in the blood plasma. In vitro analysis of the immunomodulatory activity of GEV-HSP70 showed that antitumor response in GEV-HSP70-treated mice was associated with the accumulation of CD8+ cells. **Conclusions**: These results demonstrate the high feasibility and efficacy of the new technique based on HSP70 encapsulated in plant vesicles in activation of the specific response to colon tumors.

## 1. Introduction

Immunotherapy is now the method of choice for a great number of cancers, and almost all the techniques employ the ability of specific molecules to enhance the antitumor response of a patient. Some of such molecules serve as adjuvants that are able to recognize tumor-associated antigens and participate in their presentation by dendritic cells. This function is typical for HSP70 molecular chaperone that was convincingly proven to elicit antigen-presenting activity leading to the activation of both innate and adaptive immune response [1,2]. It is well established that HSP70 or its particular peptides linked to tumor antigens work efficiently in numerous anti-cancer vaccine constructs whose efficacy has been established in pre-clinical and clinical trials [3,4,5,6,7,8,9,10,11]. Notably, pure recombinant HSP70 alone was employed in cell and animal tumor models and demonstrated the ability to generate a powerful immune response towards melanoma tumors [12,13]. In our studies, human recombinant HSP70 was also employed to increase the recognizability of C6 glioblastoma cells in vitro and in vivo [14], which was probably related to the ability of the chaperone to bind the phosphatidylserine component of the plasma membrane and penetrate living cells [15,16]. It was found that HSP70 entering melanoma and colon cancer cells displaced its intracellular counterpart and expulsed the latter being soluble or embodied in exosomes; in the latter form, HSP70 demonstrated greater immunogenic and antitumor activity [17,18,19]. Thus, delivery of pure HSP70 to a tumor could potentially lead to activation of both adaptive and innate antitumor immune responses.

The use of extracellular vesicles as carriers is a promising approach for the delivery of therapeutic biomolecules. Given that the use of animal-derived exosomes is hampered by ethical and manufacturing restrictions, plant-derived vesicles have been increasingly considered as effective delivery vehicles for exogenous therapeutic biomolecules in recent years [20,21,22]. Plant-derived extracellular vesicles are nanosized particles secreted by plant cells, surrounded by a lipid bilayer, and containing proteins, lipids, and nucleic acids. It is believed that plant vesicles mainly mediate the response of plant cells to abiotic stress and pathogen infection [20,21,22]. A few studies have successfully attempted to use intact plant vesicles from cabbage and ginger to deliver therapeutic biomolecules [23,24,25], including ours, which showed that grapefruit vesicles can effectively deliver exogenous recombinant HSP70 to cultured mammalian cells while maintaining its functional activity [26]. The data prompted us to use the HSP70 protein inserted in plant vesicles obtained from grapefruit which were demonstrated to hold considerable amounts of the active chaperone [26]. Thus, the aim of this work was to test the possibility of using grapefruit vesicles to deliver exogenous HSP70 to colon tumor cells in vivo with subsequent analysis of the activation of antitumor immunity.

## 2. Materials and Methods

### 2.1. Recombinant HSP70 Preparation

Synthesis of human recombinant HSP70 protein was carried out in *E. coli* bacteria transfected with pMS-Hsp70 plasmid (presented by Prof. R. Morimoto, Evanston, IL, USA). The obtained BL21 DE3 bacterial strain was grown on LB medium at 37 °C to OD = 0.5. HSP70 expression was induced by 1 mM IPTG followed by co-incubation for two hours, after which the bacteria were harvested and lysed at −80 °C freezing after 30 min incubation at room temperature in a buffer: 0.5 mM ethylenediaminetetetraacetic acid (EDTA), 50 mM Na_3_PO_4_, pH 8.5 with 1 mg/mL lysozyme. The protein was purified by sequential two-step chromatography. Bacterial lysates were passed through a DEAE-Sepharose column (GE Healthcare UK Ltd., Little Chalfont, UK) equilibrated with buffer A [20 mM NaCl, 20 mM Tris-HCl, 0.1 mM EDTA, pH 7.5], after which bound proteins containing Hsp70 were eluted from the column with buffer A containing 0.5 M NaCl. The eluate was dissolved in buffer B [20 mM Tris-HCl, 2 mM MgCl2, pH 7.5] and passed through ATP-agarose (Sigma-Aldrich, St Louis, MO, USA). HSP70 was eluted with 15 mM ATP, added up to 5 mM EDTA, and precipitated with ammonium sulfate followed by centrifugation. The precipitate was dissolved in phosphate buffer (PBS) and subjected to dialysis using Pierce Slide-a-Lyser cassettes (Pierce, Rockford, IL, USA). The resulting HSP70 solution was further purified by incubation with polymyxin B-agarose gel (Sigma-Aldrich, St Louis, MO, USA) and sterilized by passing through a 0.2 mm pore diameter filter (Millipore, Burlington, MA, USA). Protein concentration was measured according to the Bradford method.

### 2.2. Isolation of Vesicles from Fruit Parts of Citrus × Paradisi (Grapefruits) and Loading of Grapefruit-Derived Vesicles with Proteins

For the isolation of grapefruit-derived extracellular vesicles (GEV), the sequential ultracentrifugation method was applied, as described earlier [26]. The juice was obtained using a household citrus juicer from supermarket-purchased grapefruits. The obtained juice was successively centrifuged (JA-10 rotor, Avanti J-25I Centrifugation, Beckman Coulter, Brea, CA, USA) at 1500× *g*, 3000× *g*, 16,000× *g*, and 10,000× *g* for 30 min, three times for 20 min, 1 h and 15 h, respectively, after which the supernatant was ultracentrifuged (Ti45 rotor, Optima L-90K Ultracentrifuge, Beckman Coulter, Brea, CA, USA) at 4 °C at 150,000× *g* for 2 h, followed by resuspension of the gel pellet in PBS overnight at 4 °C. The pellets were then broken mechanically with a pipette, diluted with PBS, and centrifuged at 16,000× *g* for 1 h (SW55 rotor, Optima L-90K Ultracentrifuge, Beckman Coulter, Brea, CA, USA) for additional purification. The collected supernatant was again ultracentrifuged for 2 h at 150,000× *g* (SW55 rotor, Optima L-90K Ultracentrifuge Beckman Coulter, Brea, CA, USA) to concentrate the vesicles. The resulting transparent pellets were diluted with PBS and incubated overnight at 4 °C. The resulting grapefruit vesicle preparations were then flash-frozen in liquid nitrogen and stored at −80 °C for further experiments.

The size of GEVs and their concentration in suspensions were determined by Nanoparticle Tracking Analysis (NTA) using the NanoSight LM10 (Malvern Instruments, Worcestershire, UK) analyzer, equipped with a blue laser (45 mW at 405 nm) and a C11440-5B camera (Hamamatsu Photonics K.K., Fukuoka City, Japan). Recording and data analysis were performed using the NTA software 2.3. The following parameters were evaluated during the analysis of recordings monitored for 60 s: the average hydrodynamic diameter, the mode of distribution, the standard deviation, and the concentration of vesicles in the suspension.

A combination of passive and active cargo loading was used. Recombinant human HSP70 protein at a final concentration of 0.1 mg/mL was mixed with suspension of GEVs at a final concentration of ~3 × 10^12^ particles/mL and incubated overnight at 4 °C. Then, the mixture was sonicated at a frequency of 35 kHz for 15 min at RT by the Bandelin SONOREX SUPER ultrasonic bath (Bandelin Electronic GmbH & Co. KG, Berlin, Germany) at room temperature and incubated for an additional 90 min at 4 °C. To remove the excess free protein, the vesicles were purified using ultrafiltration through a 100-kDa filter (Amicon, Millipore, Temecula, CA, USA) ten times with washing by PBS. The obtained suspension of HSP70-loaded grapefruit vesicles (GEV-HSP70) was adjusted to the starting volume of the initial suspension of GEVs with PBS and sterilized by filtration through a 0.22 µm filter (Millipore, Temecula, CA, USA). The final concentration of loaded GEVs was established by NTA.

### 2.3. Evaluation of the Efficiency of the GEV Loading by Western-Blotting

The HSP70 protein amount in the samples of GEV-HSP70 was determined by western blotting. The purified samples of GEV-HSP70 were incubated at 4 °C for 30 min with 20 µL of lysis buffer (7M urea, 2M thiourea, 4% CHAPS, 1% DTT). The same number (2 × 10^11^) of vesicles isolated from grapefruit (without loading procedure) was analyzed in parallel. Recombinant HSP70 at 0.2, 0.5, 1.0, and 2.0 μg per lane was also analyzed by Western blotting. The protein samples were diluted in Laemmli buffer (BioRad, Hercules, CA, USA), subjected to 10% SDS-PAGE containing 0.1% SDS, and transferred to the PVDF membrane (Thermo Scientific, Waltham, MA, USA) using the Trans-Blot Turbo Transfer System (BioRad, Hercules, CA, USA). Immunoblotting was performed according to the Blue Dry Western protocol [27]. Mouse monoclonal antibodies to HSP70 (clone 8D1, patent # Ru2722398) were used as primary antibodies at 1:500 dilution. Horseradish peroxidase-conjugated rabbit anti-mouse polyclonal antibodyies (Cloud-Clone Corp., Wuhan, China) were used as secondary antibodies at 1:5000 dilution. Chemiluminescent detection of the protein bands was performed with Clarity Western ECL Blotting Substrate (Bio-Rad, Hercules, CA, USA) and ChemiDoc System (BioRad, Hercules, CA, USA). Aliquots of the recombinant HSP70 (0.2 to 2 μg per lane) were used in densitometry for subsequent evaluation of the amount of protein loaded into GEV-HSP70.

### 2.4. Cryo-Electron Microscopy Evaluation of the Efficiency of the GEV Loading Using Western-Blotting

Direct visualization of the grapefruit-derived vesicles and loaded GEVs was performed by Cryo-EM as described previously [26,28]. The aqueous solution of the sample was applied on a glow-discharged lacey carbon EM grid, which was then plunge-frozen into the precooled liquid ethane with Vitrobot Mark IV (ThermoFisher Scientific, Waltham, MA, USA). The samples were studied using a cryo-electron microscope Titan Krios 60-300 TEM/STEM (ThermoFisher Scientific, Waltham, MA, USA), equipped with TEM direct electron detector Falcon II (ThermoFisher Scientific, Waltham, MA, USA) and Cs image corrector (CEOS, Heidelberg, Germany) at accelerating voltage of 300 kV. To minimize radiation damage during image acquisition, low-dose mode in EPU software (ThermoFisher Scientific, Waltham, MA, USA) was used. The resulting micrographs of GEVs were analyzed using the open-source image analysis and processing program ImageJ 1.54g (National Institutes of Health, Bethesda, MD, USA).

### 2.5. Cells

HCT116 and DLD1 human colon cancer cells were obtained from the Cell Culture Collection, Institute of Cytology of the RAS (St. Petersburg, Russia). Mouse colon carcinoma CT-26 cells were kindly provided by Prof. G. Multhoff (Technical University of Munchen, Germany). Cultured CT-26 cells were stably transfected with pHIV-iRFP720-E2A-Luc plasmid as previously described [17]. The resulting CT26_iRFP720-E2A-Luc_ cells expressed near far-red fluorescent protein (ex. 698 nm/em. 720 nm) and luciferase. To assess the activation of the antitumor immune response in vitro, a culture of natural killer (NK) cells was used. NK-92 cells were kindly provided by Dr. Elena Kovalenko (Shemyakin-Ovchinnikov Institute of Bioorganic Chemistry, RAS (Moscow, Russia).

HCT116 and DLD1 cells were cultured in DMEM-F12 (BioLot, St. Petersburg, Russia) containing 10% heat inactivated fetal bovine serum (FBS) and CT-26 or CT-26_iRFP720-E2A-Luc_ cells were grown in RPMI-1640 media supplemented with 10% FBS (HyClone, Logan, UT, USA), 2 mM L-glutamine, 100 U/mL penicillin and 0.1 mg/mL streptomycin (PanEco, Moscow, Russia) in a 5% CO_2_ atmosphere with 90% humidity. The number and viability of cells were estimated on a LUNA-II™ Automated Cell Counter (Logos Biosystems, Dongan-gu, Republic of Korea) after mixing the cell suspension with trypan blue (1:1).

### 2.6. Cytotoxicity Assay

The cytotoxicity assay was performed with the xCELLigence RTCA system (Agilent, Santa Clara, CA, USA). This impedance-based assay carries out label-free, real-time, high-throughput analysis of cell growth and lymphocyte-mediated cytotoxicity [29]. Since NK cells and other lymphocytes are non-adhesive, they do not possess impedance [29]; therefore, a change in electrical signal relates only to adherent tumor cells. To evaluate the sensitivity of cancer cells (incubated with soluble recombinant HSP70 or HSP70 in the composition of GEVs) to cytotoxic lymphocytes, intact CT-26 mouse colon cancer cells and HCT116 or DLD1 human colon cancer cell were seeded in the wells of an E-plate at concentrations of 5 × 10^3^ cells/well and incubated during 18 h at standard condition. Then HSP70 (50 μg/mL or 5 μg/mL), or the GEVs loaded with recombinant HSP70 (0.5 × 10^12^ GEVs/mL, concentration of loaded HSP70 ~2.5 μg/mL), or GEVs without loading (0.5 × 10^12^ GEVs/mL), were added. 18 h later, effector cells isolated from the spleen of C3HA mice (3 × 10^5^ cells/well) or human NK-92 cells (1 × 10^6^ cells/well) were added with the exchange of culture medium, and recording was carried out over the next 48 h. Each experimental point was duplicated within one experiment. All experiments were performed in triplicate.

### 2.7. Animal Experiments

All in vivo experiments were carried out following the requirements of the Institute of Cytology of the Russian Academy of Sciences Ethic Committee (Identification number F18-00380). Male C3HA mice were obtained from the Scientific and Production Enterprise “Nursery of Laboratory Animals” of the Institute of Bioorganic Chemistry of the Russian Academy of Sciences (Pushchino, Russia). BALB/c mice were purchased from the Biomedical Technology Research Center (Nizhniy Novgorod, Russia).

Male BALB/c mice were used for subcutaneous CT-26 tumor formation. The mice were divided into 4 groups (18 animal/group) and subcutaneously injected with 2 × 10^5^ CT26_iRFP720-E2A-Luc_ cells (hereafter referred to as CT-26) per mouse. The cells were previously mixed with: (i) cultural medium (“Untreated” group); (ii) HSP70 (50 µg/mouse, “HSP70” group); (iii) GEVs loaded with HSP70 (4 × 10^11^ loaded vesicles/mouse, quantity of loaded HSP70 ~2 μg/mouse, “GEV-HSP70” group); (iiii) GEVs (4 × 10^11^ vesicles/mouse, “GEV” group).

Tumor formation in animals of the experimental groups was assessed every 3 days starting from the eighth day after the injection of CT-26 cells using direct measurement of the tumor node with a caliper. The tumor volume was estimated using the formula $V=L\times D\times \frac{D}{2}$, where L is the length of the largest dimension, and D is the width or the smallest dimension.

Tumor growth rate was also estimated by weighing tumors taken from 8 control and treated animals on day 21 after engrafting; blood and spleens were collected on the same day. Tumors were photographed and weighed. Blood plasma was frozen at −80 °C before cytokine measurement by ELISA, while spleens were used immediately. Five random CT26_iRFP720-E2A-Luc_-injected mice from each group were subjected to bioimaging with the use of the IVIS Spectrum imaging system (Perkin-Elmer, Beaconsfield, UK) on day 21.

The lifespan of four experimental groups of animals, each consisting of 10 mice, was assessed daily for 3 months, with the fact of death of each animal recorded. Survival curves were established according to the method of Kaplan-Meier and compared using a Mantel-Cox method.

To estimate the specific cytotoxic activity, the splenocytes of mice from all experimental groups were used. For the precise analysis of the total or specific CD8+ cell response, we first isolated spleen cells from animals belonging to appropriate treatment groups and further divided each into two groups. One group was incubated with Dynabeads FlowComp™ Mouse CD8 (Invitrogen, Carlsbad, CA, USA) to isolate CD8+ cells, and the other comprised the total lymphocyte fraction. These cell populations were used as effector cells, which were added to CT-26 cells at a ratio of 100:1. Cell viability was analyzed using the xCELLigence equipment as described above.

The collected blood of 5 animals from each group was centrifuged for 1 h at 3000× *g* at +4 °C to obtain blood plasma. The level of cytokines TGFB-1 and IL-10 in the blood plasma of experimental animals was assessed using the ELISA Kit for Transforming Growth Factor Beta 1 (TGFb1) (Cloud-Clone Corp., Wuhan, China) and ELISA Kit for Interleukin 10 (IL10) (Cloud-Clone Corp., Wuhan, China) according to the manufacturer’s instructions. All the probes were triplicated.

### 2.8. Statistics

Visualization and analysis of the obtained data were carried out in GraphPad Prism 9.5.1 software. For multiple comparisons of group means, one-way ANOVA with Tukey’s test was used. To process data on the survival of experimental animals, the Kaplan-Meier estimate was used. Analysis of the results of western blotting and Cryo-EM was carried out using the freely available software ImageJ 1.54g. Data are presented as mean ± SD.

## 3. Results

### 3.1. Characterization of Native and HSP70-Loaded GEVs

**Figure 1:**
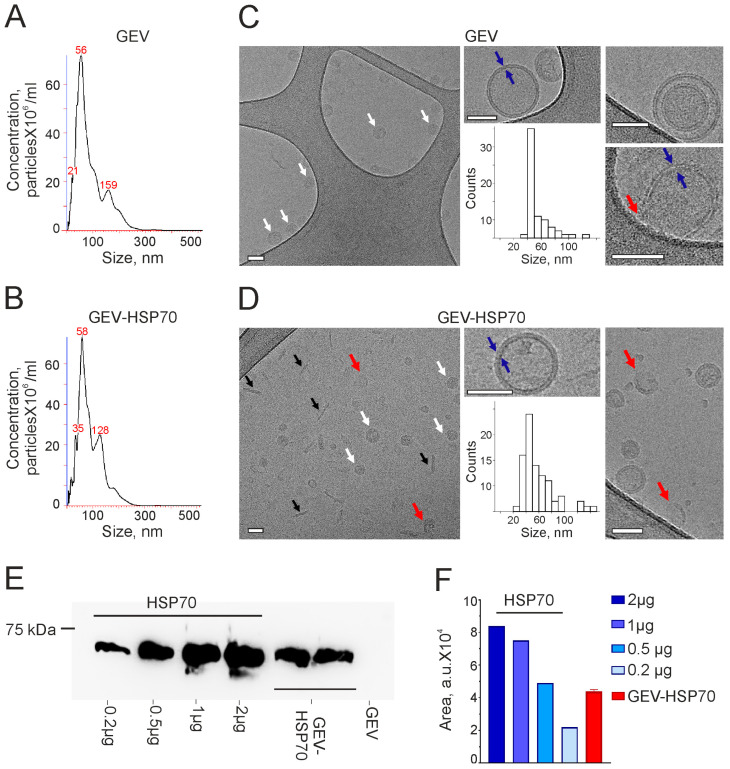
Size, concentration, and morphology of grapefruit-derived vesicles before (GEV) and after loading procedure (GEV-HSP70). (**A**,**B**) Typical examples of nanoparticle tracking analysis (NTA) of the sample of isolated GEV (**A**) and GEV-HSP70 (**B**). (**C**,**D**) Cryo-EM images of GEV (**C**) or GEV-HSP70 (**D**). White arrows indicate vesicles with intact membrane, red arrows indicate vesicles with broken membrane, and black arrows—debris in the sample. The blue arrows depict a lipid bilayer membrane of the vesicle. Scale bars are 50 nm. Inset–size distribution histogram. A total of 100 particles were analyzed. (**E**,**F**) Loading efficiency of GEV with HSP70 protein: (**E**) Example of Western blot (WB) of HSP70 in the initial GEV (line 7) and GEV-HSP70 (lines 5,6) in an amount of 10^11^ particles per line. Recombinant HSP70 in an amount from 0.2 μg to 2 μg per line (lines 1–4). (**F**) Cumulative quantification of HSP70 loaded into GEV obtained from WB.

First, we analyzed the particle concentration and size of GEVs as well as their morphology and integrity before and after the loading procedure. Using NTA method, we showed that there were no significant changes in the median particle size after loading. The particles had a median size of 58 ± 7 nm before and 56 ± 6 nm after the loading (Figure 1A,B), but the concentration of particles decreased by a factor of 1.5–2 times.

The study using Cryo-EM demonstrated that native GEVs have a predominantly spherical shape, surrounded by a lipid bilayer with an average particle size of 53 ± 20 nm. Double vesicles and particles with impaired membrane integrity were occasionally encountered (Figure 1C). The GEV-HSP70 showed the appearance of some contaminant debris in the sample, as well as an increase in the number of vesicles with impaired integrity. At the same time, the average size of the analyzed particles did not change and was 55 ± 18 nm (Figure 1D). Thus, using the NTA and Cryo-EM methods, it was shown that the loading procedure does not significantly change the overall morphology and size of GEVs.

The loading efficiency of human recombinant HSP70 into GEVs was quantified using WB with anti-HSP70 antibodies (Figure 1E,F). Lysed GEV-HSP70 in an amount of 10^11^ particles/lane was applied to the WB, as well as rHSP70 in an amount from 0.2 μg to 2 μg per lane. Using ImageJ software, we established a linear standard curve (R = 0.92), according to the equation of which it was found that 10^11^ particles contain approximately 0.5 μg of protein. Thus, in all animal experiments, the administered 4 × 10^11^ GEV-HSP70- corresponded to about 2 μg of recombinant HSP70 per dose, and in vitro experiments used approximately 0.5 μg/well but not more than 1 μg/well of the protein.

### 3.2. Recombinant HSP70 Loaded into GEVs Effectively Stimulates Immune Cell Activity In Vitro

Recently, we have demonstrated that mouse exosomal HSP70, as well as a soluble HSP70, were able to pull out intracellular chaperones on cancer cell surfaces and activate the cytotoxic response of natural killer (NK) cells [12]. In this study, we tested the immunomodulatory activity of GEV-HSP70 in several colon cancer models.

**Figure 2:**
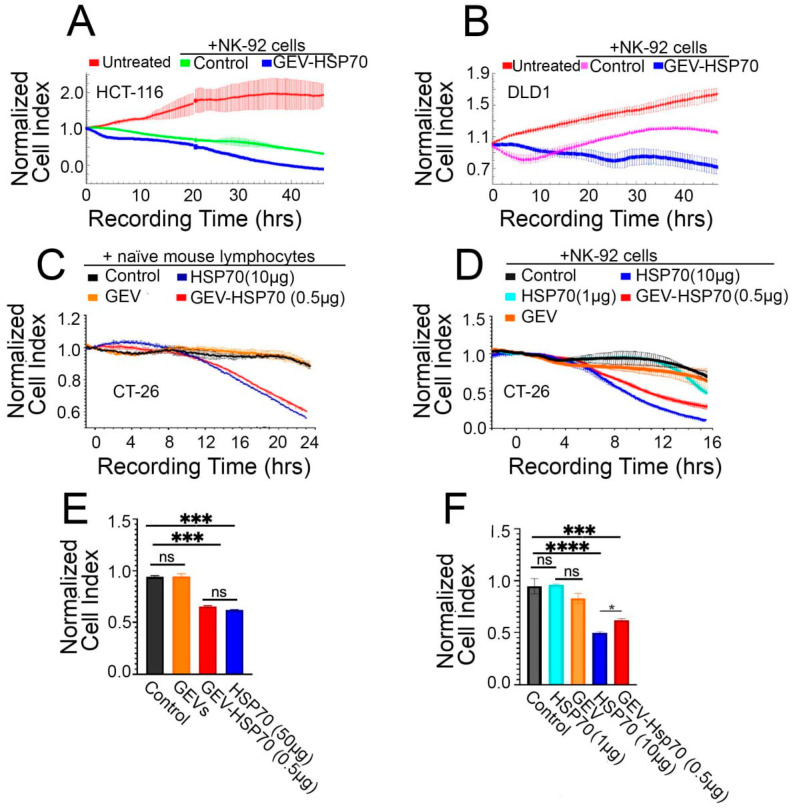
Effect of free HSP70 and GEV-HSP70 on the proliferative activity of human and murine colon cancer cells when attacked by cytotoxic lymphocytes (CTL) from naïve C3HA mice or human NK-92 cells. (**A**,**B**) GEV-HSP70 increases the sensitivity of human colon cancer cells to the NK cells action: HCT-116 (**A**) and DLD1 (**B**) cells were seeded to wells of E-plate and incubated or not with GEV-HSP70 for 24 h, and then NK-92 cells were added into wells. Recoding on xCELLigence equipment lasted 45 h. (**C**–**F**) Effect of free and GEV-loaded HSP70 on the proliferative activity of CT-26 cells when attacked by CTL from naïve C3HA mice or human NK-92 cells. (**C**,**E**) Proliferative activity of CT-26 cells preincubated with HSP70 (10 μg), GEV (0 μg HSP70), and GEV-HSP70 (about 0.5 μg HSP70) when exposed to the CTL. (F,H). Proliferative activity of CT-26 cells preincubated with HSP70 (10 μg or 1 μg), GEV (0 μg HSP70), and GEV-HSP70 (about 0.5 μg HSP70) upon exposure to NK-92 cells. Addition of rHSP70, GEV-HSP70, and naïve GEVs at 24 h of incubation, addition of effector cells at 40 h of incubation. For the (**E**,**F**) panels, the last observation time point was chosen for analysis. In panels (**A**–**D**), the normalization point for the proliferation curves is chosen to correspond to the time point of effector cells. Pairwise multiple comparisons were performed using ANOVA with Tukey’s posterior test. A statistically significant difference between the is indicated as **** for *p* < 0.0001, *** for *p* ≤ 0.0005, * for *p* < 0.05, ns—not statistically significant.

At first, to test whether GEV-HSP70 is also capable of sensitizing cancer cells to cytotoxic cells, we used two human colon carcinoma cells, HCT-116 and DLD1, incubated with GEV-HSP70, in cytotoxic T lymphocyte (CTL) assay using xCELLigence technique. It is shown that the pre-incubation of human colon cancer cells with GEV-HSP70 resulted in a 2-3-fold reduction of cell index, which indicated an increase in the sensitivity of tumor cells to cytotoxic NK-92 cells (Figure 2A,B).

Then, we compared the immunomodulatory activity of GEV-HSP70 and HSP70 in mouse CT-26 colon carcinoma cells in vitro. CT-26 cells were preincubated with rHSP70 (10 μg/well), as well as with GEV-HSP70 (10^11^ particles/well, containing about 0.5 μg of rHSP70), then naïve lymphocytes obtained from the spleens of C3HA mice (Figure 2C) or NK-92 cells (Figure 2D) were added and cytotoxic activity was monitored in real-time using the xCELLigence technique. We observed an increase in the toxic effect of CTL both to cells incubated with HSP70 in free form or GEV-HSP70 (Figure 2C,E). Of note, the effect was equal for cells that were co-incubated with free HSP70 (10 μg), the amount of which was 20 times higher than in GEV-HSP70 (about 0.5 μg of HSP70). A similar result was obtained when NK-92cells were used. The effect of natural killer cells was observed after 10 h of co-incubation, and cell survival decreased by 50% (Figure 2D,F). To test the assumption that accumulation of rHSP70 in mouse cells is more efficient when the protein is encapsulated in vesicles, free rHSP70 was also previously added to the cells in an amount of 1 μg, which approximately corresponds to its content in GEV-HSP70 samples. It was shown that the amount of free HSP70 comparable to that loaded in GEVs does not lead to NK cell activation, as do unloaded GEVs (Figure 2D,F).

Thus, we have shown that the addition of HSP70 in free form and as part of GEVs increases the sensitivity of human (HCT-116, DLD1) or mouse (CT-26) colon cancer cells to mouse cytotoxic lymphocytes and human NK-92 cells. Moreover, as part of GEVs, HSP70 caused activation of antitumor immunity when the amount of protein was 20 times less as compared to the protein added in free form.

### 3.3. Antitumor Effect of HSP70 and GEV-HSP70 in a Mouse Model of Colorectal Cancer

Next, we analyzed the effect of GEV-HSP70 on the tumor growth of CT-26 cells in vivo. A single administration of the GEV, GEV-HSP70 (2 μg HSP70/dose), and HSP70 (50 µg) were used at the same time as tumor cells were inoculated subcutaneously to male Babl/c mice.

**Figure 3:**
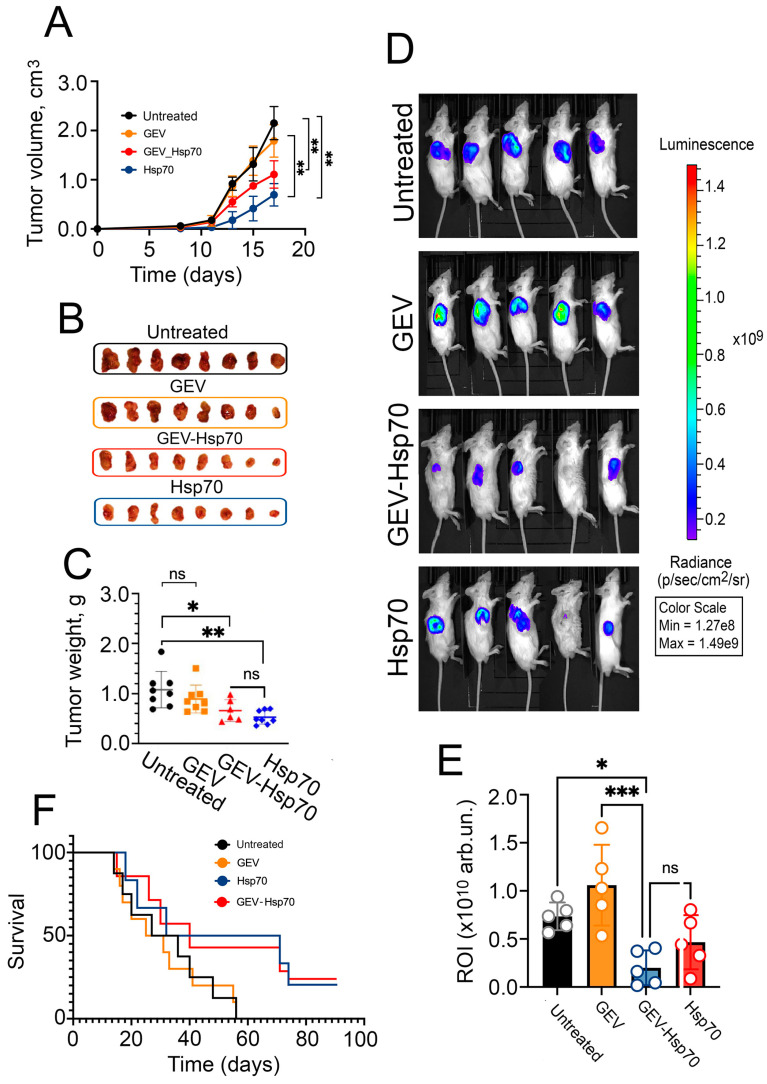
Antitumor effect of HSP70 and GEV-HSP70 in a mouse model of colorectal carcinoma. (**A**) Analysis of the growth rate of the tumor node during the 21st day of observation in 4 groups of animals after inoculation of CT-26 cells (Untreated) or CT-26 cells mixed with GEV, GEV-HSP70 (2 μg HSP70/dose), and with HSP70 (50 μg/dose) (N = 8) (**B**,**C**) Twenty-one days after CT-26 cells inoculation tumors were isolated, photographed (**B**) and weighed (**C**) (N = 8). (**D**,**E**) Analysis of the tumor size by the intravital luminescence imaging system (N = 5). (**F**) Life expectancy of animals in control and experimental groups (N = 10). Legend *** for *p* ≤ 0.0005, ** for *p* ≤ 0.005, * for *p* < 0.05, ns—not statistically significant.

For three weeks after inoculation of tumor cells, either intact or in the presence of GEV, HSP70, or GEV-HSP70, we measured the volume of growing tumors. We observed a delay in tumor growth in the “HSP70” and ‘GEV-HSP70′ groups; their average size on the last day of measurement was significantly lower than the size in the “Untreated” group or in the “GEV” group (2.0 ± 0.2 for “GEV-HSP70” or 1.0 ± 0.4 for “HSP70” groups vs 3.4 ± 0.6 and 2.7 ± 0.4 cm^3^ for “Untreated” and “GEV” groups respectively) (Figure 3A).

Then, tumors from eight animals from each group were isolated (Figure 3B) and weighed (Figure 3C). Again, the weight of tumors from the “Untreated” group or from the “GEV” group varied significantly from the “HSP70” and “GEV-HSP70” groups (0.5 ± 0.3 and 0.5 ± 0.2 g for “GEV-HSP70” and “HSP70” respectively vs. 1.1 ± 0.4 and 0.9 ± 0.3 g for “Untreated” and “GEV” groups respectively). It was also shown that the average tumor luminescence in “GEV-HSP70” was 4.0-fold less than in the “Untreated” group and 5.8-fold less than in the “GEV” group. The luminescence of tumors from the “HSP70” group was not statistically different from that in the “GEV-HSP70” group (Figure 3D,E).

Animal survival of ten remaining mice in each group was monitored over a 90-day period, which showed a 3-fold increase in lifespan for animals in the “HSP70” and “GEV-HSP70” groups compared to the control groups (Figure 3F). The average life span in the two control groups, “Untreated” and “GEV”, was 32.3 ± 5.4 and 29.8 ± 4.8 days, respectively, while in the “GEV-HSP70” and “HSP70” groups, 3 mice in each group survived the observation time (90 days), and the remaining had average lifespan 42.2 ± 9.6 and 45.9 ± 9.0 days.

The data obtained indicate that the delivery of HSP70 into CT-26 tumor cells as part of grapefruit vesicles leads to a decrease in the tumor growth as well as, tumor weight, or size, as well as an increase in the life expectancy of experimental animals compared to the control group with the same efficiency, as free rHSP70 in 25-fold exceeding quantities.

### 3.4. Activation of a Specific Immune Response in Animals Received HSP70 and GEV-HSP70 in a Mouse Model of Colon Carcinoma

**Figure 4:**
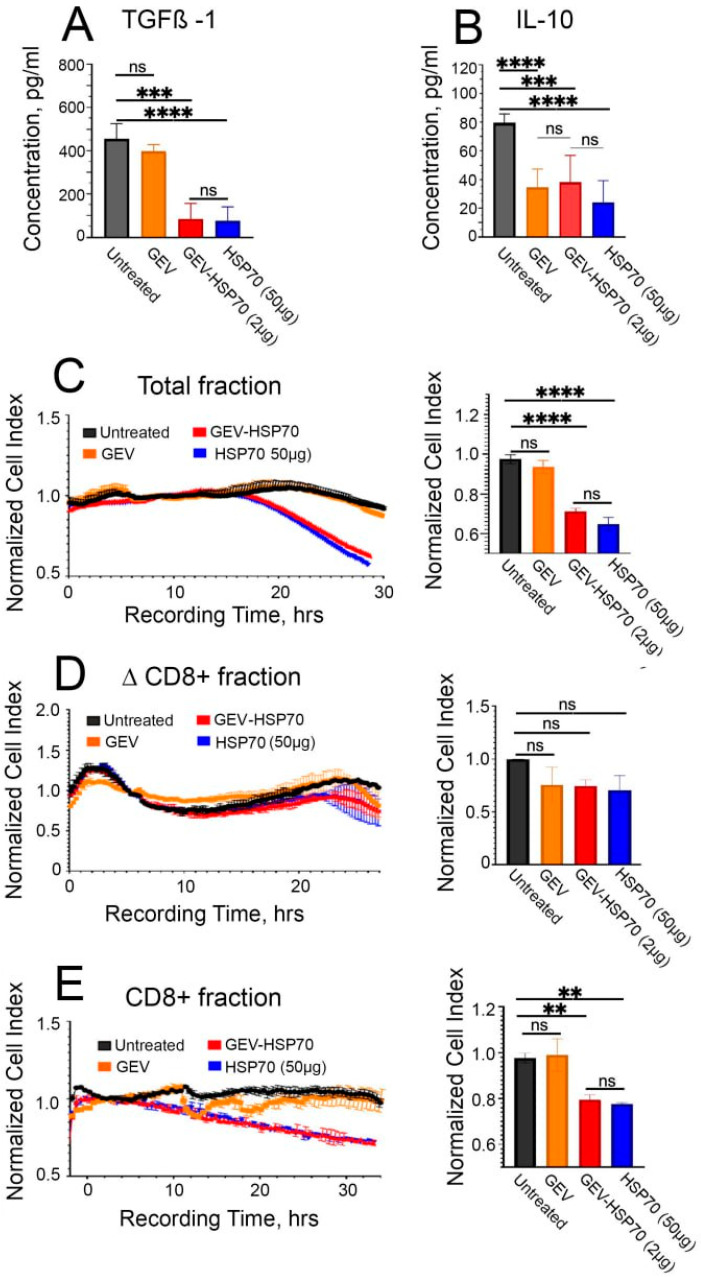
GEV-HSP70 and free HSP70 induce a specific antitumor immune response in the CT-26 mouse model of colorectal carcinoma. (**A**,**B**) Assessment of the concentration of cytokines IL-10 and TGFB-1 in the blood plasma of mice 21 days after inoculation with CT-26 cells (Untreated), CT-26 cells mixed with GEV, GEV-HSP70 (about 2 μg HSP70/dose) or HSP70 (50 μg/dose) (N = 5). (**C**) Proliferative activity of CT-26 cells when exposed to the total fraction of lymphocytes isolated from the spleens of mice of experimental and control groups. (**D**) The influence of the lymphocyte fraction depleted of CD8+ T-lymphocytes on the proliferative activity of CT-26 cells. (**E**) Cytostatic effect of CD8+ T lymphocytes from mice from experimental and control groups on the proliferation of CT-26 cells (N = 5). Pairwise multiple comparisons were performed using ANOVA with Tukey’s posterior test. *p* < 0.0001 = ****, *p* ≤ 0.0005 = ***, *p* ≤ 0.005 = **, ns—not statistically significant.

To analyze if the immune response was possibly stimulated in animals by HSP70 administration, blood samples were collected on the 21 days of tumor growth, followed by ELISA assay for TGFβ-1 and IL10. It was shown that in the blood of mice of the “HSP70” and “GEV-HSP70” groups, there was a significant reduction in the levels of IL-10 and TGFβ-1 compared to the “Untreated” group (Figure 4A,B). It is also worth noting that in the “GEV” group, we observed a 2-fold decrease in the level of pro-inflammatory IL-10 in the blood.

In order to further verify that the observed in vivo antitumor effects are related to the activation of a specific immune response, the proliferative activity of CT-26 cells was assessed during their co-incubation with the total fraction of lymphocytes obtained from the spleens of mice of experimental and control groups, as well as during co-cultivation of CT-26 cells with a fraction of cytotoxic CD8+ T lymphocytes obtained from the total lymphocyte fraction using the DynabeadsFlowComp™ Mouse CD8 kit. Cell viability and proliferative activity were assessed using the xCellLigence system. It was shown that after the addition of total lymphocytes or CD8+ T cells obtained from HSP70 or GEV-HSP70 groups of mice, CT-26 cell viability was reduced by 30% and 20%, respectively (Figure 4C,E). Lymphocyte fractions depleted of CD8+ cells, for which no stimulatory or cytotoxic effect was observed, were also used as a control (Figure 4D). The data obtained indicate that specific CD8+ cytotoxic T lymphocytes are involved in the observed in vivo antitumor effect of HSP70, both in free form and loaded into GEVs.

## 4. Discussion

Multifaceted function of HSP70 chaperone in tumor growth and immune response to cancer is well established, and there are hundreds of publications dedicated to this topic [30]. In addition to its protective activity, HSP70 was shown to leave cancer cells or enter them; when occurring exogenously, HSP70, by binding tumor antigens or a variety of other polypeptides, is able to regulate immune response to cancer cells by triggering the mechanisms of innate and adaptive immunity [2].

HSP70 is released from tumor cells in free form or within extracellular vesicles (EV) [2,31]. HSP70-containing EVs or exosomes strongly affect tumor progression by promoting the activity of Tregs or inducing myeloid-derived suppressor cells, which help the tumor avoid immune surveillance [32,33]. On the other hand, HSP70-containing EVs from heat-treated CT-26 and B16 melanoma cells exhibited antitumor activity, which seemed to be associated with the enhancement of a strong Th1 immune response [4]. Exosomes from mouse colon carcinoma MC38 cells also showed strong antitumor effects associated with the conversion of regulatory T cells into Th17 cells [34]. Our earlier data demonstrated that EVs from tumor cells loaded with HSP70 caused high antitumor immune responses in mouse B16 melanoma and CT-26 colon carcinoma-bearing mice, activating CD8+ dependent immune response [17]. Taking into account that tumor exosomes may be dangerous to apply as a therapeutic agent, we loaded grapefruit vesicles with pure HSP70 and found that they efficiently activated innate pro-tumor immunity to colorectal cancer cells in vitro slowed down tumor growth and increased the survival rate in the animal model in vivo, stimulating specific antitumor CD8+ dependent immunity, which was accompanied by a reduction in the amount of pro-tumor cytokines, TGFβ-1 and IL-10.

## 5. Conclusions

Summarizing the results, we can conclude that the HSP70 protein activates the antitumor immune response in the models of colorectal cancer, both in vitro and in vivo. Recombinant HSP70 protein can be loaded into grapefruit vesicles while maintaining its functionality. Moreover, in both cell and animal models, HSP70 in the composition of GEVs has the same antitumor effect as a 20-fold greater amount of the free form of the chaperone.

## Data Availability

No additional data available.
